# Analysis and prediction of unforced errors in men’s and women’s professional padel

**DOI:** 10.5114/biolsport.2024.134763

**Published:** 2024-03-06

**Authors:** Rafael Conde-Ripoll, Diego Muñoz, Bernardino J. Sánchez-Alcaraz, Adrián Escudero-Tena

**Affiliations:** 1 Universidad Europea de Madrid, Spain; 2 University of Extremadura, Spain; 3 University of Murcia, Spain

**Keywords:** Racquet sports, Performance analysis, Shot efficacy, High performance, Gender

## Abstract

The aims were to I. assess the differences in unforced errors between men’s and women’s professional padel players, II. assess the differences in unforced errors between winners and losers of the set in professional padel players, III. analyse the evolution of unforced errors in professional padel. To do this, the unforced errors (which are provided by World Padel Tour (WPT)) were collected from 2,567 sets (1,476 men’s sets and 1,091 women’s sets) corresponding to matches from the final draw (round of 32, round of 16, quarterfinals, semi-finals and finals) of tournaments on the WPT men’s and women’s circuits during 2016 to 2022 seasons, which are available on the WPT YouTube channel and on the WPT TV website. The results with respect to the first aim indicate that the number of unforced errors was higher in women’s padel than in men’s, regardless of the set number, tournament round, season and court. With respect to the second aim, not committing unforced errors is a fundamental factor in professional padel to win the sets. Finally, regarding the third aim, the number of unforced errors decreased as the seasons progressed; therefore, to win, players should not only have to pass the ball without missing, but they might have to hit winners or generate forced errors of the opponent.

## INTRODUCTION

Padel is a racket sport played in pairs on a 20 × 10 metre court, which is divided by a central net, with an exterior four-metre-high glass and netting enclosure at the back of the court and a lateral three-metre enclosure to the sides, against which the ball can be hit during play [1]. Padel is practised by players of different ages and competitive levels due to the simplicity of its rules and the fact that its physical and technical-tactical demands are adapted to the level of play [2–4]. This sport is currently played in more than 70 countries [1], which in recent years has led to a significant increase in the number of facilities, commercial agreements (sponsorships, employment contracts, etc.), sports licences, etc. [1]. In this context, the number of scientific studies on padel has increased in recent years [5, 6], with the most studied topic being analysis of performance.

In professional padel there are several tournament circuits (A1-Padel Tour, Premier Padel, World Padel Tour (WPT)), with the WPT being most important; it organises more than 20 events in different cities around the world each season. Thus, the players on this circuit have been the subject of several research studies that have identified the differences that exist in the parameters of play between men’s and women’s padel [7–10]. Studies have found that the duration of the points is longer in women’s padel, as well as the number of shots per point [7, 8]. In terms of shot types, men hit more backhand volleys, flat or topspin smashes, and shots close to the net, while women hit more lobs, trays, and shots from the middle zone or the back of the court [8–12]. In addition, women make a higher percentage of errors (forced and unforced), while men make a higher percentage of winners [13, 14]. On the other hand, although men are more effective on the serve [15], women are more effective on break points [10, 16].

Similarly, studies have been conducted to identify the differences between match-winning and match-losing professional padel pairs [17, 18]. These studies indicate that match-winning pairs typically win long points (over 11 seconds), do not make unforced errors in the first four seconds of the point, and are very effective on break points. In addition, they perform more attacking actions in 85% of the points, spend more time in the net areas and hit more smashes. On the other hand, match-losing pairs tend to lose longer points, perform fewer attacking actions per point and per match, hit more groundstrokes with or without the wall during the match and hit more trays. Thus, a relationship has been found between winning points and playing in areas close to the net [19].

The last shot of the point has been widely analysed in men’s and women’s professional padel [10, 19–21] as well as in other racket sports such as tennis or badminton [22–26]. These studies have concluded that the point can end with a winning shot, a forced error or an unforced error. A winning shot is that action where a player wins the point with a direct shot, while a forced error is that action where the player loses the point due to an error in a highly difficult shot, and with a poor position for its execution due to the opponent’s prior shot [21]. And, finally, an unforced error is that action where the player loses the point due to bad play or an error in a situation that should be fully controlled by the player [19].

It is worth noting that the majority of points in professional padel and tennis matches end as a result of unforced errors [20, 27]. Moreover, making fewer unforced errors than the opponent is key for attaining success in padel [10, 19–21], tennis [28–30] and badminton [25, 31, 32]. Specifically, in padel, in relative terms, unforced errors usually occur with the double wall and, in absolute terms, with the forehand, backhand and volley [20].

Therefore, after carrying out an analysis of the scientific literature in professional padel and even in other racket sports, unforced errors seem to be a fundamental performance indicator. To the best of our knowledge, this is the first study which takes into account data from seven seasons. For this reason, the aims of the current study were: I. to assess the differences in unforced errors between men’s and women’s professional padel players, II. to assess the differences in unforced errors between winners and losers of the set in professional padel players, III. to analyse the evolution of unforced errors in professional padel.

## MATERIALS AND METHODS

### Samples

A total of 2,567 sets (1,476 men’s sets and 1,091 women’s sets) corresponding to matches from the final draw (round of 32, round of 16, quarterfinals, semi-finals and finals) of tournaments on the WPT men’s and women’s circuits were analysed. The data collection was carried out from the recording of the unforced errors provided by the WPT during the 2016 to 2022 seasons at the end of each set in the open access videos of WPT TV (https://www.worldpadel-tourtv.com/) or on the official YouTube channel (https://www.youtube.com/@Worldpadeltour), following the ethical provisions of the Declaration of Helsinki [33].

### Study variables

The performance indicator analysed was the unforced errors (as used in other investigations [17, 19, 20]). According to WPT, an unforced error occurs when a pair loses the point due to bad play in a situation without pressure from the opponents that should be fully controlled by the player of the team [19].

In addition, the following contextual variables were established: gender of the players (men and women), result of the set (winners or losers), tournament round (round of 32, round of 16, quarterfinals, semi-finals, and finals), set number (first, second and third sets) and court (outdoor and indoor).

With respect to the “result of the set” variable, several previous investigations have studied aspects of scoring in professional padel [34, 35]. These investigations use the set as a unit of measurement, instead of the match, since the data vary very significantly depending on whether two or three sets are played. According to the rules [1], a padel match is won by the pair that wins two sets before the opponent. Therefore, in the scenario of a three-set match, the results could lead to confusion as each pair would win and lose a set before playing a third and final set.

### Process

The unforced errors analysed (which are provided by WPT) were collected from matches that are available on the WPT YouTube channel and the WPT TV website. The responsible analyst who recorded the unforced errors in WPT during the seasons indicated in the sample was a certified padel coach with more than 10 years of experience. However, an inter-observer reliability analysis was performed to ensure the veracity of the data collected. A doctor in sports sciences, author of numerous relevant scientific publications on padel and with more than 10 years of experience, using the LINCE video analysis software [36], analysed the unforced errors of a random sample of 380 sets to guarantee a relevant amount of data, between 10 and 20% of the total study sample [37]. The reliability of the analysis test was 0.93, considered almost perfect [38]. This doctor collected the contextual variables of the study, through an ad-hoc instrument. Furthermore, once the registration was completed, the doctor again collected a random sample of 380 sets in order to perform an intra-observer reliability analysis, with the average reliability of the analysis test being 0.98, considered almost perfect [38]. These reliability procedures have already been used in relevant padel studies [10, 17, 39, 40].

### Statistical analysis

Unforced errors did not meet the normality criteria (Kolmogorov-Smirnov), so non-parametric statistical methods were used [41]. A descriptive analysis (means and standard deviations) of unforced errors organised according to the contextual variables was carried out. Subsequently, an inferential analysis was conducted, using the Mann-Whitney U test to identify differences between men’s and women’s professional padel and between the winners and losers of the set in professional padel according to the set number, the round, the season and outdoor or indoor tournament. Effect size was calculated, considering a small (0.20), medium (0.50), and large (0.80) effect size [42]. In addition, graphs were created through the Holt prediction model to determine the evolution of unforced errors in professional padel. Statistical analyses were performed using SPSS v.21 software (IBM Corp., Armonk, NY, USA) and statistical significance was set at p < 0.05.

## RESULTS

Table 1 shows the differences found between men’s and women’s professional padel in unforced errors according to the set number, tournament round, season and court.

**TABLE 1:** Unforced errors as a function of the winning or losing pair of the set by gender (data expressed as mean and standard deviation)

Unforced errors according to:		Winners						Losers					

		Men		Women		p	d	Men		Women		p	d
	
		X	SD	X	SD			X	SD	X	SD		
Set	First	6.12	3.26	7.46	3.76	**< 0.001**	0.369	7.78	3.31	9.44	3.88	**< 0.001**	0.435
	Second	6.01	3.23	7.21	3.53	**< 0.001**	0.341	7.97	3.39	8.98	3.61	**< 0.001**	0.291
	Third	6.05	3.27	6.92	3.39	**0.004**	0.316	7.76	3.34	9.08	3.63	**0.001**	0.381

Round	Round of 32	5.36	2.83	6.32	3.18	**0.021**	0.310	7.36	3.52	7.99	3.16	**0.049**	0.265
	Round of 16	5.46	2.72	6.03	2.89	0.118	0.208	6.50	2.66	7.54	2.65	**0.016**	0.324
	Quarterfinals	6.04	3.25	7.43	3.63	**< 0.001**	0.380	8.01	3.37	9.37	3.82	**< 0.001**	0.345
	Semi-finals	6.51	3.44	7.54	3.73	**< 0.001**	0.271	8.02	3.29	9.53	3.79	**< 0.001**	0.404
	Finals	6.12	3.33	7.78	3.75	**< 0.001**	0.482	8.18	3.45	9.84	3.92	**< 0.001**	0.482

Season	2016	7.54	3.55	7.93	3.66	0.340	0.118	9.09	3.46	10.73	3.83	**0.001**	0.409
	2017	6.44	3.15	8.04	3.93	**< 0.001**	0.440	8.79	3.20	10.11	4.19	**0.007**	0.322
	2018	7.31	3.72	8.53	4.11	**0.020**	0.300	10.17	3.78	10.54	3.59	0.238	0.151
	2019	7.13	3.45	9.19	4.08	**< 0.001**	0.519	9.35	2.98	11.15	4.27	**< 0.001**	0.482
	2020	5.93	3.04	6.83	3.38	**0.021**	0.278	7.07	3.33	8.51	3.65	**0.001**	0.389
	2021	4.85	2.81	6.13	3.11	**< 0.001**	0.429	6.49	2.81	7.88	3.17	**< 0.001**	0.440
	2022	5.25	2.76	6.51	3.03	**< 0.001**	0.428	6.78	2.75	8.24	3.06	**< 0.001**	0.501

Court	Outdoor	6.22	3.37	7.49	3.59	**< 0.001**	0.376	8.22	3.57	9.56	3.51	**< 0.001**	0.412
	Indoor	6.01	3.21	7.22	3.63	**< 0.001**	0.341	7.73	3.26	9.08	3.80	**< 0.001**	0.349

X: mean; SD: standard deviation; p: p-value; d: effect size.

As shown in Table 1, professional women’s padel players committed significantly more unforced errors than men (p < 0.001, p = 0.001, p = 0.004, p = 0.007, p = 0.020, p = 0.021, p = 0.016 or p = 0.049), regardless of the set number, tournament round, season or type of court, except for the winners in the round of 16 (p = 0.118) and in the 2016 season (p = 0.340), where although women committed more unforced errors than men, these differences were not significant. Furthermore, in the losers in the 2018 season, men committed more unforced errors than women, although this difference was not significant (p = 0.238).

**FIG. 1:**
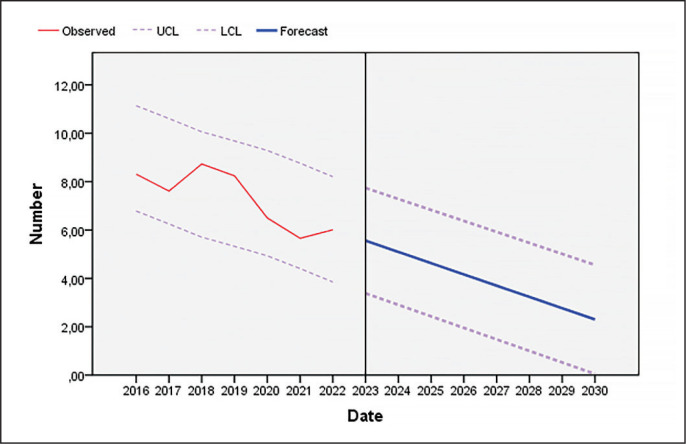
Evolution of unforced errors in men’s professional padel until the year 2030 (UCL: upper confidence limit; LCL: lower confidence limit).

**FIG. 2:**
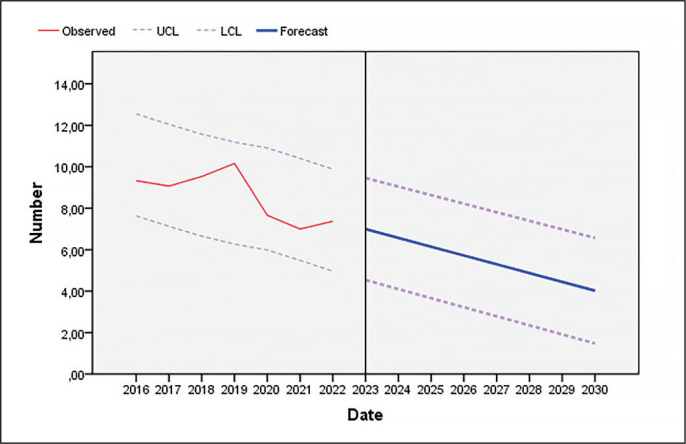
Evolution of unforced errors in women’s professional padel until the year 2030 (UCL: upper confidence limit; LCL: lower confidence limit).

As Figures 1 and 2 show, the number of unforced errors decreased as the seasons progressed in both men’s professional padel and women’s professional padel.

Table 2 shows the differences found between winners and losers in men’s and women’s professional padel in unforced errors according to the number set, tournament round, season and court.

**TABLE 2:** Descriptive and inferential analysis of unforced errors as a function of gender by winning/loser pair

Unforced errors according to:		Men						Women					

		Winners		Losers		p	d	Winners		Losers		p	d
	
		X	SD	X	SD			X	SD	X	SD		
Set	First	6.12	3.26	7.78	3.31	**< 0.001**	0.532	7.46	3.76	9.44	3.88	**< 0.001**	0.533
	Second	6.01	3.23	7.97	3.39	**< 0.001**	0.612	7.21	3.53	8.98	3.61	**< 0.001**	0.515
	Third	6.05	3.27	7.76	3.34	**< 0.001**	0.569	6.92	3.39	9.08	3.63	**< 0.001**	0.610

Round	Round of 32	5.36	2.83	7.36	3.52	**< 0.001**	0.623	6.32	3.18	7.99	3.16	**< 0.001**	0.546
	Round of 16	5.46	2.72	6.50	2.66	**< 0.001**	0.455	6.03	2.89	7.54	2.65	**< 0.001**	0.543
	Quarterfinals	6.04	3.25	8.01	3.37	**< 0.001**	0.618	7.43	3.63	9.37	3.82	**< 0.001**	0.530
	Semi-finals	6.51	3.44	8.02	3.29	**< 0.001**	0.467	7.54	3.73	9.53	3.79	**< 0.001**	0.556
	Finals	6.12	3.33	8.18	3.45	**< 0.001**	0.661	7.78	3.75	9.84	3.92	**< 0.001**	0.545

Season	2016	7.54	3.55	9.09	3.46	**< 0.001**	0.484	7.93	3.66	10.73	3.83	**< 0.001**	0.748
	2017	6.44	3.15	8.79	3.20	**< 0.001**	0.777	8.04	3.93	10.11	4.19	**< 0.001**	0.546
	2018	7.31	3.72	10.17	3.78	**< 0.001**	0.765	8.53	4.11	10.54	3.59	**< 0.001**	0.580
	2019	7.13	3.45	9.35	2.98	**< 0.001**	0.677	9.19	4.08	11.15	4.27	**< 0.001**	0.459
	2020	5.93	3.04	7.07	3.33	**0.002**	0.352	6.83	3.38	8.51	3.65	**0.001**	0.464
	2021	4.85	2.81	6.49	2.81	**< 0.001**	0.607	6.13	3.11	7.88	3.17	**< 0.001**	0.584
	2022	5.25	2.76	6.78	2.75	**< 0.001**	0.587	6.51	3.03	8.24	3.06	**< 0.001**	0.576

Court	Outdoor	6.22	3.37	8.22	3.57	**< 0.001**	0.597	7.49	3.59	9.56	3.51	**< 0.001**	0.630
	Indoor	6.01	3.21	7.73	3.26	**< 0.001**	0.564	7.22	3.63	9.08	3.80	**< 0.001**	0.511

X: mean; SD: standard deviation; p: p-value; d: effect size.

As shown in Table 2, the results show that the players who won the set committed significantly fewer unforced errors than the players who lost the set (p < 0.001 or p = 0.002), regardless of the set number, tournament round, season or court type.

**FIG. 3:**
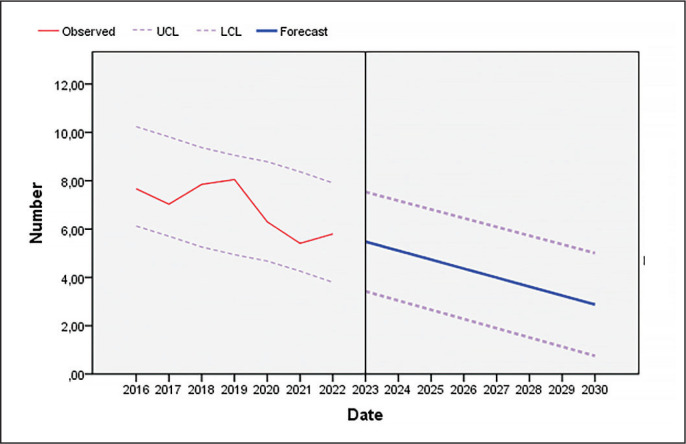
Evolution of unforced errors committed by players who win sets in professional padel until the year 2030 (UCL: upper confidence limit; LCL: lower confidence limit).

**FIG. 4:**
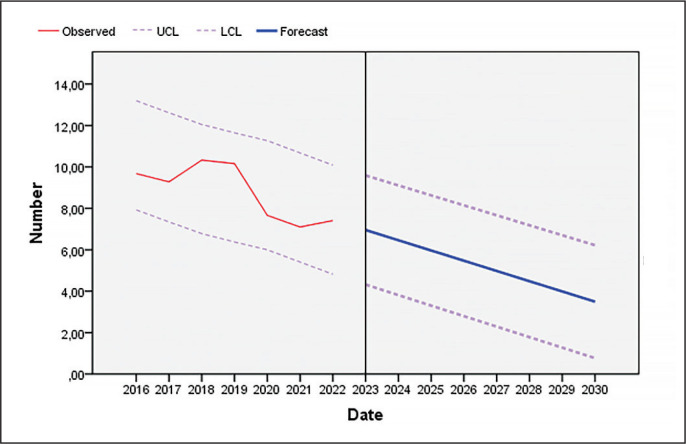
Evolution of unforced errors committed by players who lose sets in professional padel until the year 2030 (UCL: upper confidence limit; LCL: lower confidence limit).

As Figures 3 and 4 show, the number of unforced errors decreased as the seasons progressed, both in the pair of players who won the sets and in the pair that lost them.

## DISCUSSION

The aims of the current study were to: I. assess the differences in unforced errors between men’s and women’s professional padel, II. assess the differences in unforced errors between winners and losers of the set in professional padel, III. analyse the evolution of unforced errors in professional padel.

The style of play in professional padel varies according to the gender of the athletes. The results of this study indicate that the number of unforced errors was higher in women’s padel than in men’s padel, regardless of the set number, tournament round, season, and court. Various studies that have analysed the most frequent technical-tactical action in professional men’s and women’s professional padel, as well as the differences between them, agree with the results of this study [13, 14]. Apart from showing that a higher number of errors (forced and unforced) are committed in women’s compared to men’s padel, it has also been found that men produce more winners than women. Women’s professional padel is characterized by a more conservative style of play, where more continuity shots such as lobs and trays are performed [8, 9, 11, 12] and where to win, it is enough to pass the ball and not commit unforced errors. In contrast, in men’s professional padel fewer unforced errors are committed and the athletes make more aggressive shots such as flat or topspin smashes [12, 14] to perform winning shots or to generate forced errors of the opponent. Nevertheless, these differences between playing styles in professional padel according to the gender of the athletes could be due to anthropometric and strength differences between elite men’s and women’s players [4, 43], since men are on average taller, and have a higher muscle percentage and higher values of vertical jump and grip strength than women players, which allows them to play a more aggressive game.

Not committing unforced errors is a fundamental factor in professional padel to win the sets. The results of this study show that the players who won committed fewer unforced errors than the players who lost the set, regardless of the game context analysed. Hence, to enhance their chances of success in matches, athletes should work closely with their coaches to develop strategies that minimize unforced errors during practice. For instance, coaches and players could watch competitive or practice matches, identify when unforced errors occur and find out ways to improve [44], and likewise, coaches could also propose exercises in which the unforced errors are monitored and penalized [45, 46]. Further investigation in this domain is required, given the scarcity of information regarding the influences of specific drills and training protocols in enhancing technical-tactical abilities [47].

As shown in Figures 1–4, players reduced the number of unforced errors between 2016 and 2022. This could be due to the professionalization of the sector which has led to enhancements in player skills. In addition, according to the data of the present investigation, the number of unforced errors will decrease as the seasons progress; therefore, to win, players must not only pass the ball, but must also hit winners or generate forced errors of the adversary. Various studies that have analysed the effectiveness of the last shot of the points in professional men’s and women’s padel in different game contexts [13, 17, 18, 48, 49] show that the players who win the matches commit fewer errors (forced and unforced) and more winners than the players who lose, which would reinforce one of the main objectives of this sport, which is to minimize the number of errors (forced and unforced). In addition, depending on the type of shot, the players who lose perform a higher percentage of backhand volleys [8, 18] and off-the-wall shots [8, 48]. Coaches and athletes should work in a cooperative way so that the latter develop a greater competitive performance by being more diverse and unpredictable in their actions thanks to appropriate challenging constraints [50]. Likewise, previous studies have indicated that winning pairs tend to win long points (more than 11 seconds) and do not usually commit unforced errors in the first four seconds of the point [19]. The same authors found that, at the net, losers committed unforced errors earlier in the rally than winners whereas there were no significant differences at the baseline. Similarly, Courel-Ibáñez and collaborators [51] found that winners made 40% fewer unforced errors at the net compared to losers. The study conducted by Escudero-Tena and collaborators [14] indicated that in men’s padel more winning shots are made than in women’s padel; thus men’s padel is generally more aggressive, although this difference decreases as the importance of the point increases. On the other hand, in men’s padel fewer errors (forced and unforced) are committed than in women’s padel at non-key moments and at key moments, so women must train to achieve a more fluid game. However, men commit more errors (forced and unforced) than women in golden points, with women being more effective in these cases. Likewise, Sánchez-Alcaraz and collaborators [34] stated that the importance of the point for the match score causes players to change their game behaviour. Therefore, players must pay special attention to the different moments of the game, and create training routines in which specific situations are established, that is, train with a scoreboard simulation.

The results of this study are very novel, because as far as we know, it is the first study that analyses such an extensive data sample (seven seasons), allowing a predictive analysis of the development of the number of unforced errors in men’ and women’s professional padel. However, a limitation must be taken into account when interpreting the results, which is that the data recorded for the round of 32 and round of 16 pertain only to the 2022 season. In previous seasons, WPT did not provide these data in these or prior rounds (i.e., rounds from qualification). In addition, in future research, as unforced errors have been analysed, forced errors, winners, and shots that generate forced errors should be analysed.

## CONCLUSIONS

The style of play in professional padel varies according to the gender of the athletes, because the number of unforced errors is higher in women’s padel than in men’s, regardless of the number of sets, the round of the tournament, the season and the court. Hence, since the number of unforced errors in men’s and women’s professional padel will decrease as the seasons progress, the style of play in women’s padel game should evolve so as to be more offensive.

In addition, not committing unforced errors is a fundamental factor in professional padel to win sets. Thus, players who win commit fewer unforced errors than those who lose, regardless of the context of the game. However, the number of unforced errors will decrease as the seasons progress. Therefore, in order to increase the chances of winning, players will not only have to pass the ball, but they will also have to hit winners and/or force errors on the opponent.
